# MRI for Differentiation between HPV-Positive and HPV-Negative Oropharyngeal Squamous Cell Carcinoma: A Systematic Review

**DOI:** 10.3390/cancers16112105

**Published:** 2024-05-31

**Authors:** Linda L. Chen, Iris Lauwers, Gerda Verduijn, Marielle Philippens, Renske Gahrmann, Marta E. Capala, Steven Petit

**Affiliations:** 1 Department of Radiotherapy, Erasmus MC Cancer Institute, University Medical Center Rotterdam, 3015 GD Rotterdam, The Netherlandsg.verduijn@erasmusmc.nl (G.V.); m.capala@erasmusmc.nl (M.E.C.);; 2 Department of Radiotherapy, University Medical Center Utrecht, 3584 CX Utrecht, The Netherlands; 3 Department of Radiology and Nuclear Medicine, Erasmus Medical Center, 3015 GD Rotterdam, The Netherlands; r.gahrmann@erasmusmc.nl

**Keywords:** oropharyngeal squamous cell carcinoma, systematic review, human papillomavirus, head and neck carcinoma, magnetic resonance imaging, apparent diffusion coefficient

## Abstract

**Simple Summary:**

Human papillomavirus-positive (HPV+) oropharyngeal squamous cell carcinoma (OPSCC) has a different disease course compared to HPV-negative (HPV−) OPSCC. This systematic review aims to investigate whether magnetic resonance imaging (MRI) can discriminate between HPV+ and HPV− OPSCC or predict HPV status in OPSCC patients using MRI. Our results show that parameters derived from structural MRI and diffusion-weighted MRI are able to discriminate between HPV+ and HPV− cases and predict HPV status with reasonable accuracy. Other MRI sequences have yet to prove their added value for the discrimination and prediction of HPV status in OPSCC patients. Machine learning studies that compared predictive models with and without clinical variables found that performance improved significantly when clinical variables were included in the model. Before the clinical implementation of MRI for HPV status determination, larger studies with external model validation using independent datasets are needed.

**Abstract:**

Human papillomavirus (HPV) is an important risk factor for oropharyngeal squamous cell carcinoma (OPSCC). HPV-positive (HPV+) cases are associated with a different pathophysiology, microstructure, and prognosis compared to HPV-negative (HPV−) cases. This review aimed to investigate the potential of magnetic resonance imaging (MRI) to discriminate between HPV+ and HPV− tumours and predict HPV status in OPSCC patients. A systematic literature search was performed on 15 December 2022 on EMBASE, MEDLINE ALL, Web of Science, and Cochrane according to PRISMA guidelines. Twenty-eight studies (*n* = 2634 patients) were included. Five, nineteen, and seven studies investigated structural MRI (e.g., T1, T2-weighted), diffusion-weighted MRI, and other sequences, respectively. Three out of four studies found that HPV+ tumours were significantly smaller in size, and their lymph node metastases were more cystic in structure than HPV− ones. Eleven out of thirteen studies found that the mean apparent diffusion coefficient was significantly higher in HPV− than HPV+ primary tumours. Other sequences need further investigation. Fourteen studies used MRI to predict HPV status using clinical, radiological, and radiomics features. The reported areas under the curve (AUC) values ranged between 0.697 and 0.944. MRI can potentially be used to find differences between HPV+ and HPV− OPSCC patients and predict HPV status with reasonable accuracy. Larger studies with external model validation using independent datasets are needed before clinical implementation.

## 1. Introduction

Human papillomavirus (HPV) status has been recognized as one of the most important risk factors for oropharyngeal squamous cell carcinoma (OPSCC) [1,2]. HPV-positive (HPV+) cases are associated with a different pathophysiology, microstructure, and prognosis compared to HPV-negative (HPV−) cases [1,2]. Moreover, patients with HPV+ tumours generally respond better to radiation treatment and have lower mortality rates than patients with HPV− tumours [1,3]. From a histopathological perspective, HPV− OPSCC are more often keratinizing and are more likely to have more maturing squamous differentiation [4]. In fact, the differences between HPV+ and HPV− tumours are so pronounced that they are regarded as separate disease classes with different TNM staging [5,6]. In spite of these differences, both HPV− and HPV+ patients receive the same treatment [5,7,8]. The current curative standard of treatment for locally advanced OPSCC is an intense chemoradiation regimen causing considerable unwanted side effects [9,10]. As the prevalence of HPV+ OPSCC increases, it becomes crucial to investigate the possibility of treating HPV+ tumours with a less aggressive approach to reduce treatment-related toxicity [5,7,11].

Because of the differences in pathophysiology, prognosis, and treatment response, it is essential to determine the HPV status of the tumour before the start of treatment. The current gold standard method to determine HPV status is by performing HPV polymerase chain reaction (PCR) on biopsied material [12,13]. Although PCR for HPV determination is accurate, it is also costly, time-consuming, and invasive [12,13]. Because of these disadvantages, p16 immunohistochemistry is often applied as a surrogate marker, as it is more accessible, less expensive, and faster to perform [14]. However, p16 immunohistochemistry has a false positive rate of about 20% and requires an invasive biopsy as well [12,15,16].

As an alternative to invasive tests, imaging modalities have been considered to determine HPV status. Compared to invasive tests, magnetic resonance imaging (MRI) has several advantages: it is non-invasive, which lowers the treatment burden, and MRI is applied routinely in the diagnostic work-up and radiotherapy treatment planning of OPSCC, which means that the workflow only needs to be minimally adjusted [17,18]. This means that MRI has the potential to provide an independent assessment of HPV status, with a negligible addition of time and cost. Hence, there is increasing interest in investigating whether MRI can differentiate between HPV+ and HPV− tumours [19,20,21,22,23]. Apart from the structural MRI sequences (e.g., T1-, T2-weighted), other sequences such as Dynamic Contrast Enhanced (DCE) and Diffusion-Weighted Imaging (DWI) are also worth investigating, as these sequences provide information about the differences in microstructure and tissue perfusion between HPV+ and HPV− tumours [2,24,25,26,27,28,29]. Moreover, applications such as radiomics and machine learning are also interesting to investigate, as these techniques can be applied to find features through histogram or textural analysis that would not have been uncovered otherwise [21,22,30,31,32].

Although there is growing interest in the usage of MRI to determine HPV status in OPSCC patients, a comprehensive systematic review that compiles all relevant literature has not been published yet. Therefore, the aim of this study was to report on the literature that uses MRI to discriminate between HPV+ and HPV− cases and predict HPV status in patients with OPSCC.

## 2. Materials and Methods

### 2.1. Search Strategy

A computerized search was conducted of EMBASE, MEDLINE ALL, Web of Science, and Cochrane databases on 15 December 2022, to identify original articles published up to December 2022. This review was performed in accordance with the preferred reporting items for systematic reviews (PRISMA) guidelines and has not been registered [33]. The search terms were “OPSCC”, “MRI”, “HPV”, and their synonyms (the full search strategy for reproducing the search can be found in Supplementary Data S1).

### 2.2. Eligibility Criteria

The search results were screened and only studies were selected that focused on OPSCC patients (primary tumour or locoregional lymph nodes), MRI as an imaging modality, and studies that report differences between OPSCC patients with and without HPV infection or studies that aim to predict HPV status. Studies were excluded if the study population consisted of fewer than twenty patients, the study was not published in English, or if the study was not performed on humans. Letters, editorials, conference abstracts, meta-analyses, consensus statements, guidelines, and (systematic) review articles were also excluded.

### 2.3. Study Selection

Title and abstract screening were performed using EndNote20 (Clarivate Analytics, Philadelphia, PA, USA) by two independent reviewers (I.L. and L.C.). Afterwards, full-text screening was performed using the eligibility criteria.

Of the included studies, I.L. and L.C. independently assessed the methodological quality of the selected studies using the QUADAS-2 tool, which assesses studies on possible bias from patient selection, index test, reference standard, and flow and timing [34]. Discussion between the reviewers was carried out for any discrepancies in the screening process and quality assessment.

### 2.4. Data Extraction and Analysis

The following data were extracted from the selected studies:

1. Study characteristics: first author, year of publication, studied subsite(s), study design, total patient number, number of HPV+ patients, number of HPV− patients, and HPV determination method (e.g., PCR, p16).
2. Used features: usage of clinical variables, structural MRI, diffusion-weighted MRI (DW-MRI), and other MRI sequences.
3. Analysis methods: study design for HPV status (descriptive/predictive) and, if predictive, type of model, type of validation, area under the receiving operating curve (AUC), accuracy, sensitivity, specificity, and significant variables.
4. DWI characteristics (if applicable): tumour delineation method (single axial image slice(s) or tumour volume), exclusion of necrotic or cystic parts (yes/no), used b-values (s/mm^2^), mean apparent diffusion coefficient (ADC_mean_) (mm^2^/s) values for HPV+ and HPV− cases with the reported *p*-value, used statistical test, and additional DWI (histogram) parameters when available.

## 3. Results

### 3.1. Search Results

**Figure 1:**
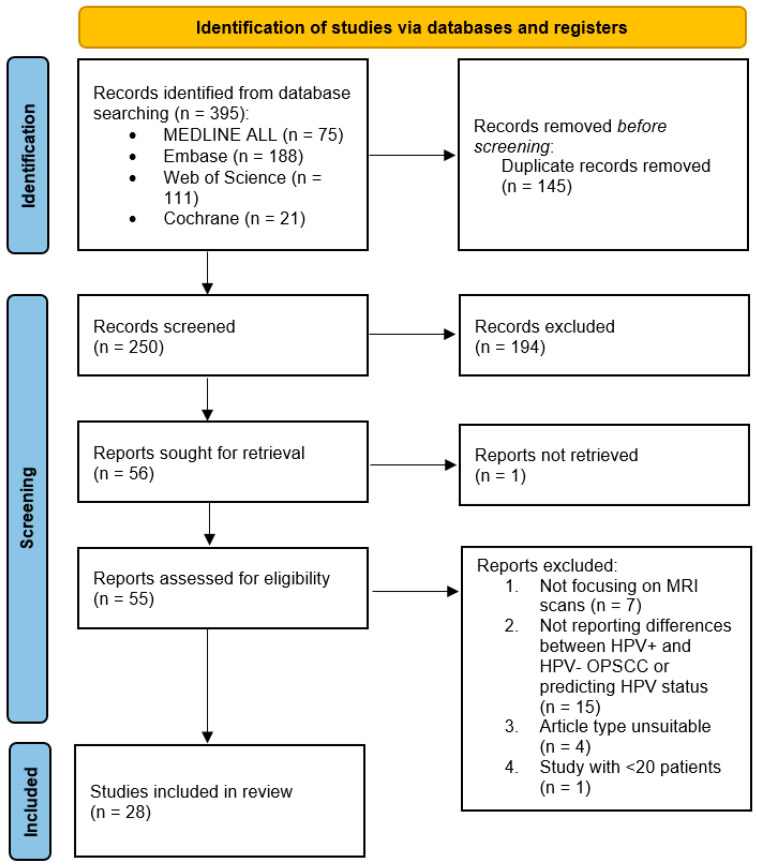
Flowchart of study selection, consistent with preferred reporting items for systematic reviews (PRISMA) statement [33].

In total, we retrieved 395 articles, of which 145 were duplicates. After the first screening of the 250 unique articles, 55 records were assessed for eligibility. Finally, 28 studies met the eligibility criteria and were included in our study (Figure 1).

### 3.2. Quality Assessment

**Figure 2:**
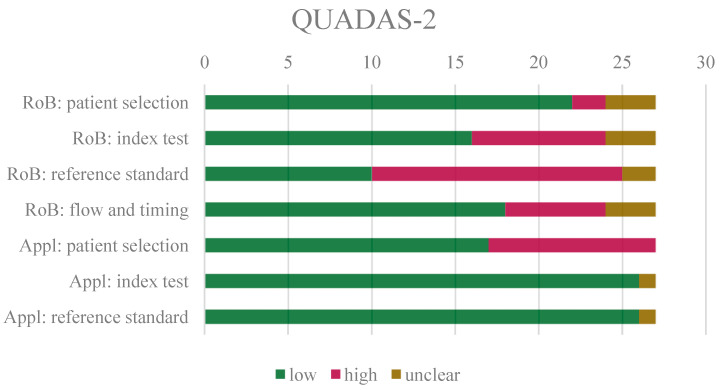
Overview of QUADAS-2 results. RoB = risk of bias; Appl = applicability.

Figure 2 summarizes the results from the QUADAS-2 tool. Overall, low risk of bias was selected most often in all assessment categories, except for the reference standard, for which high risk was most prevalent. For the reference standard, the main concern was the use of different HPV determination methods for different patients or the exclusive use of p16 immunohistochemistry for HPV determination [20,21,22,23,26,31,35,36,37,38,39,40,41,42,43,44]. The main concern for patient selection was the lack of information on consecutive or random patient selection and the exclusion of patients with lower-stage OPSCC [20,25,27,35,36,37,38,39,45,46,47]. Regarding the index test, the main concern was the lack of pre-specification of thresholds or independent validation [21,25,26,27,31,39,40,41,46,47,48]. The main concern for flow and timing was the fact that not all patients received the same reference standard or index test [21,31,32,36,37,39,40,43,48]. The QUADAS-2 assessment for each study can be found in Supplementary Data S2.

### 3.3. Study Characteristics

Twenty-eight studies with a total of 2634 patients (1571 HPV+ cases, 899 HPV− cases), conducted between 2014 and 2022, were included in this review. Fourteen out of twenty-eight studies used MRI to find differences between HPV+ and HPV− patients (Table 1) [2,19,20,25,28,29,35,38,39,41,43,47,49,50], the other studies aimed to predict HPV status using MRI (Table 2) [21,22,23,26,27,31,32,37,40,42,44,45,46,48]. The main findings from each feature category are summarized in Table 3. Twenty-one studies had a retrospective cohort study design [2,19,20,21,22,25,26,28,29,31,32,35,37,38,39,42,44,45,47,48,49], and seven studies had a prospective cohort study design [23,27,40,41,43,46,50]. Of the twenty-eight included papers, five papers focused on structural MRI sequences that were used in routine clinical examinations [19,20,21,22,23], nineteen focused on DWI sequences [2,25,27,28,29,31,32,35,36,38,39,41,42,44,45,46,47,48,49], and seven focused on other sequences (three papers had two sequences of focus) [26,27,28,35,37,40,47]. As for HPV-status determination, eleven papers used p16 immunohistochemistry exclusively [20,21,22,23,26,31,35,38,41,42,44], ten papers first used p16 immunohistochemistry and then confirmed HPV+ status using PCR [2,19,25,27,29,40,46,47,48,49], two papers used the Hybrid Capture 2 High-Risk HPV-DNA test (Qiagen, Hilden, Germany) [28,45], two articles used either p16 immunohistochemistry or in situ hybridization [43,50], one paper used either p16 immunohistochemistry or PCR [32], and two papers did not specify the HPV determination method [37,39]. Eleven studies used 1.5T MRI scanners [19,25,27,28,38,43,45,46,47,49,50], nine studies used 3.0T MRI scanners [20,22,26,29,32,37,40,41,44], and eight studies used a combination of 1.5T and 3.0T MRI scanners [2,21,23,31,35,39,42,48]. Of the twenty-eight studies, nineteen investigated OPSCC exclusively [19,20,21,22,23,27,29,31,32,37,38,40,42,44,45,46,47,48,49], three studies investigated oral cavity (OC)SCC as well as OPSCC [2,26,28], and six studies also included other tumours of the head and neck (i.e., larynx, hypopharynx, nasal, sinonasal) [25,35,39,41,43,50].

Machine learning was used in fourteen papers [21,22,23,26,27,31,32,37,40,42,44,45,46,48]. Out of the articles using machine learning, nine papers only used radiomics and radiological variables derived from MRI images [21,22,26,31,32,37,40,44,46], and five papers additionally used clinical features for the prediction of HPV status [23,27,42,45,48]. Moreover, one article investigated radiomics without using machine learning [19]. None of the models from the studies using MRI to predict HPV status were validated with an independent or external dataset. Logistic regression was the most frequently used classifier (*n* = 7) [21,22,23,27,31,32,45]. Park et al. and Suh et al. reported several classifiers to compare for their dataset [21,32]. Marzi et al. also compared different classifiers and built their final model using naive Bayes [48]. Overall, the lowest reported AUC value was 0.697 using a multivariate linear regression model, with an accompanying sensitivity and specificity of 0.769 and 0.733 [44]. The highest reported AUC value was 0.944 using a multivariate logistic regression model, with an accompanying sensitivity and specificity of 0.833 and 0.926 [45]. The models with the highest AUC values all included clinical features such as smoking, tumour sublocation, and alcohol intake (Table 1). Four studies compared the use of clinical variables with radiological variables, and all four found that a combination of clinical variables outperformed either category on its own [23,31,45,48].

### 3.4. Structural MRI Sequences

Five articles focused on structural MRI sequences, consisting of T1-weighted (T1w) non-contrast and contrast-enhanced and T2-weighted (T2w) images [19,20,21,22,23].

Three out of four studies found that HPV+ primary tumours were significantly smaller in size than HPV− primary tumours [22,23,31,48]. One study found that HPV+ primary tumours were more likely to have an exophytic appearance than HPV− primary tumours (73% vs. 63%; *p* = 0.02) [23], whilst another found a higher sphericity [31]. Moreover, HPV+ primary tumours were less likely to have ulceration and necrosis than HPV− primary tumours (10% vs. 20%; *p* = 0.002 and 9% vs. 19%; *p* = 0.002, respectively) [31].

Two studies investigated the structure of nodal metastases, and both found that cystic nodes were more prevalent in HPV+ cases compared to HPV− cases (45% vs. 32%; *p* = 0.009 and 39.4% vs. 18.5%; *p* = 0.025, respectively) [20,23]. Moreover, Chan et al. found more clustered nodes in HPV+ cases (38% vs. 28%; *p* < 0.001) [23]. Huang et al. found that necrotic nodal metastases were significantly more prevalent in HPV− than HPV+ patients (73.8% vs. 51.5%; *p* = 0.027) [20] whilst Chan et al. did not find a difference (67% vs. 63%, *p* = 0.530) [23].

Giannitto et al. investigated radiomics in structural MRI without machine learning and found the 10th and 90th percentile of the T1w intensity histogram to be significantly different between HPV+ and HPV− tumours (16.54 vs. 14.74, *p* = 0.03 and 161.02 vs. 161.57, *p* = 0.03, respectively) [19].

As for predictive studies, Sohn et al. and Park et al. used radiomics in combination with machine learning to predict HPV status using structural MRI, reporting AUC values of 0.74 and 0.83 [21,22]. First-order features like skewness, 10th and 90th percentile in the intensity histograms, and second-order features like busyness and complexity were found to be useful features for discernment between HPV+ and HPV− tumours [21,22].

### 3.5. DW-MRI Sequences

Nineteen studies made use of DW-MRI to study differences in HPV status [2,25,27,28,29,31,32,35,36,38,39,41,42,44,45,46,47,48,49]. The diffusion weighting used for DWI, referred to as b-values, can be found in Table 4. Seven out of nineteen studies used two b-values [2,28,32,38,41,42,45], while others used up to nine b-values [25,27,29,31,35,39,44,46,47,48,49,50]. The most common lowest b-value was b = 0 (*n* = 17) and the most common highest b-value was b = 1000 s/mm^2^ (*n* = 12). Seventeen studies used the ADC model, of which four mentioned the use of monoexponential fitting and the rest did not specify the type of fitting [2,27,29,46]. Two studies made use of the intravoxel incoherent motion model (IVIM) model as described by Federau et al. [27,46,51]. Six studies only delineated the tumour on one or two slices where the tumour had the largest axial diameter on DWI [2,29,38,44,45,49]. Twelve studies delineated the entire tumour volume, and one did not specify [25,27,28,31,32,35,39,41,42,46,47,48,50]. Seven out of nineteen studies excluded areas of necrosis and cysts [25,28,35,41,42,44,49].

**Figure 3:**
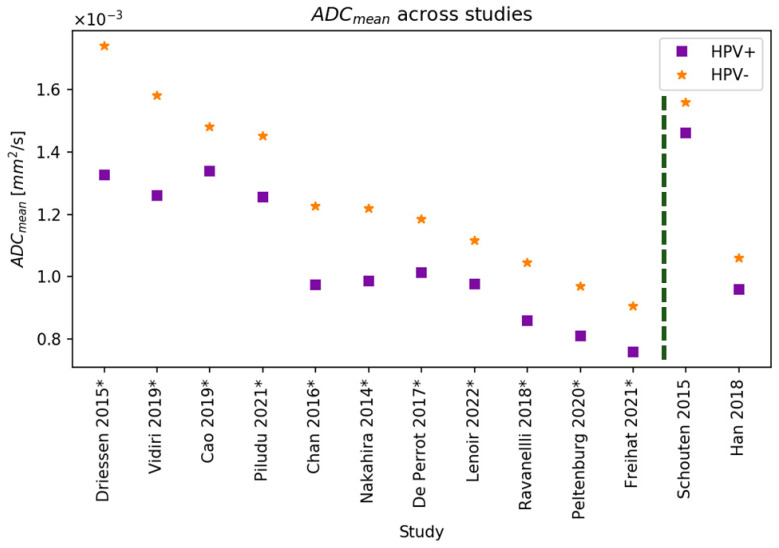
Mean apparent diffusion coefficient (ADC_mean_) of HPV+ and HPV− lesions with decreasing ADC for HPV− from left to right. If median was reported instead of mean, we used the median. Asterisks (*) denote a significant difference between HPV+ and HPV− groups [2,25,27,28,29,38,39,41,42,44,45,46,49]. A significant difference between HPV+ and HPV− lesions was found in all studies (left of the dashed line), except for Schouten et al. and Han et al. [28,49].

Eleven out of thirteen studies found that the ADC_mean_ for HPV− was significantly higher than for HPV+ OPSCC (Figure 3) [2,25,27,28,29,38,39,41,42,43,44,45,46,49]. Seven papers studied the difference in ADC_mean_ in metastatic lymph nodes, of whom Chan et al. and Piludu et al. found a significantly higher ADC_mean_ in HPV− lymph nodes compared to HPV+ lymph nodes (1.249 vs. 0.989 × 10^−3^ mm^2^/s, *p* = 0.0002 and 1.333 vs. 1.090 × 10^−3^ mm^2^/s, *p* = 0.018, respectively) [27,42]. Contrarily, five studies did not find any significant differences in the ADC_mean_ of metastatic lymph nodes [28,35,41,43,46].

Vidiri et al. and Piludu et al., using the IVIM model, found a higher tissue diffusion coefficient (D_t_) in HPV− primary tumours compared to HPV+ (1.20 vs. 0.97 × 10^−3^ mm^2^/s, *p* < 0.001 and 1.163 vs. 0.957 × 10^−3^ mm^2^/s, *p* = 0.001, respectively), while the other tested IVIM parameters were not significantly different [27,46]. Piludu et al. found a higher D_t_ for the lymph nodes in HPV− tumours (1.108 vs. 0.904, *p* = 0.005) [27]. Vidiri et al. found no differences in the IVIM-DWI parameters for the lymph nodes [46].

Five studies used DW-MRI to predict HPV status, with reported AUC values ranging from 0.77 to 0.944 [27,32,45,46,48]. Of the features derived from DW-MRI, two studies used D_t_ and one study used the ADC_mean_ from primary tumours in their model to predict HPV status [27,45,46]. Two studies investigating radiomics in MRI found that amongst the useful features were histogram parameters from ADC sequences such as the entropy and homogeneity as well as the inverse difference moment of the lymph node ADC, a measure for homogeneity [46,48].

### 3.6. Other MRI Sequences

Five studies investigated perfusion parameters from DCE-MRI to study differences in HPV status [26,27,28,35,47]. Chawla et al. made use of the shutter speed model [35]. The other four studies applied the model as described by Tofts et al. [52,53]. Three studies did not find any significant differences in the perfusion parameters obtained with DCE-MRI [27,28,35]. Choi et al. found a significantly higher volume transfer constant (K^trans^) in HPV+ tumours compared to HPV− tumours in the mean (0.23 vs. 0.14, *p* = 0.009), 25th percentile (0.18 vs. 0.10, *p* = 0.008), 50th percentile (0.22 vs. 0.13, *p* = 0.010), and 75th percentile (0.27 vs. 0.18, *p* = 0.017) [26]. Moreover, Choi et al. found that the 25th percentile of flux rate constant (k_ep_) was significantly higher for the HPV+ group than the HPV− group (0.54 vs. 0.37, *p* = 0.028) [26]. Vidiri et al. found that the histogram kurtosis of extravascular extracellular volume fraction (v_e_) was higher in HPV+ cases compared to HPV− cases (*p* = 0.009) [47].

Ahn et al. explored the application of pseudo-continuous arterial spin labelling (PCASL), where magnetically labelled flowing blood in the carotids serves as an endogenous contrast agent, eliminating the need for an injected contrast agent [40,54]. The image histogram overall standard deviation and 95th percentile of tumour blood flow (TBF_95_) were significantly different between HPV+ and HPV− cases (27.8 ± 8.7 vs. 37.7 ± 9.0 mL/100 g/min, *p* = 0.001 and 111.7 vs. 147.3 mL/100 g/min, *p* = 0.004, respectively) [40]. Ahn et al. used the standard deviation and 95th percentile of tumour blood flow from PCASL to predict HPV status, which resulted in an AUC of 0.805 after leave-one-out cross-validation tests [40].

Fujima et al. investigated the use of amide proton transfer (APT), with the hypothesis that tumours have high cellularity and may exhibit elevated APT values [37]. In their analysis, the APT coefficient of variation was significantly higher in the HPV+ group than the HPV− group (0.43 ± 0.04 vs. 0.48 ± 0.04, *p* = 0.004), though the APT mean and standard deviation were not significantly different between the two groups (*p* = 0.82 and *p* = 0.11, respectively) [37].

## 4. Discussion

In this systematic review, we aimed to report on all available literature that used MRI to find differences between HPV+ and HPV− cases and predict HPV status in OPSCC patients. For this purpose, we investigated structural sequences, DW-MRI sequences, and other MRI sequences. Our results indicate that parameters derived from structural MRI and DW-MRI show the potential to discriminate between HPV+ and HPV− tumours and predict the HPV status. On the other hand, MRI sequences DCE-MRI or ASL have yet to prove their added value to the differentiation of HPV status in OPSCC patients. Overall, none of the found predictive models have been applied yet in clinical practice.

Concerning structural MRI sequences, the findings were focused on macroscopic differences, such as the size, necrosis, and ulceration of the primary tumour or locoregional lymph nodes. As these studies used structural sequences acquired in clinical routine, the overall number of participants was highest in this category, making the findings more generalizable than the findings from other MRI sequences. However, few studies explored predictive models based exclusively on structural MRI.

In terms of findings regarding DW-MRI, ADC_mean_ was found to be significantly different between HPV+ and HPV− tumours. Despite the heterogeneity in HPV+ and HPV− group size, used b-values, delineation method, and the absolute difference in ADC_mean_, almost all studies that reported the ADC_mean_ found that it was significantly higher in the HPV− group compared to the HPV+ group. However, there were large differences in the ADC_mean_ values found within studies (Figure 3), which could be caused by the aforementioned heterogeneity of the methods. This underlines the importance of homogenizing DWI protocols across different institutes and studies. A recent study performed the optimization of b-values and identified the optimal set of b-values to maximize DWI parameter accuracy vs. scan time [55]. This set of b-values could be a good starting point to further homogenize DWI protocols across institutes. The two studies that did not find a significant difference in ADC_mean_ did not have any notable differences in sample size (±40 participants), HPV+/HPV− ratio, HPV determination method, used b-values, tumour subsites, or statistical tests compared to the other DW-MRI studies [28,49].

Of note, Chan et al. reported ADC values a thousand times larger than the other studies, which we attributed to a reporting error and corrected for [42]. As Lenoir et al. used different combinations of b-values to calculate the ADC_mean_ for HPV+ and HPV−, we decided to report the ADC_mean_ calculated from the b-values of 0 and 1000 s/mm^2^, as these were most often used in the other studies [29]. Using different combinations and different numbers of b-values, Lenoir found different levels of significance, with most combinations not yielding significant differences, highlighting the importance of using the most suitable b-values in ADC calculation [29,55]. While the findings indicate that ADC_mean_ measures tend to be significantly lower in HPV+ tumours, the diverse range of ADC_mean_ values across studies poses challenges in establishing a clinically meaningful threshold.

Several studies investigated the potential added value of other MRI sequences, such as DCE-MRI, PCASL, and APT [26,27,28,35,37,40,47]. Though there are variables that differed significantly between HPV+ and HPV− groups, further investigation is needed in larger groups before the added value of these sequences in HPV determination can be confirmed.

Machine learning studies that compared predictive models with and without clinical variables found that performance improved significantly when clinical variables were included in the model [31,48]. Amongst machine learning studies, the choice of classifiers varied, with multivariable logistic regression being the most frequently applied method [20,22,23,27,31,32,42,45]. This type of classifier performs well on smaller datasets and handles complex data better than multivariate linear regression models but is also more prone to overfitting [32,44,45,56]. Marzi et al. compared several classifiers and found that the naive Bayes performed best due to its properties of robustness in the presence of missing and noisy data and the ability to work well on small sample sizes [48,56]. Overall, validation on larger and external datasets is needed for all models before implementation in the clinic.

The clinically significant radiomics features were found to be consistent with clinical and histopathological data of both HPV+ and HPV− tumours. Bos et al. found differences in the sphericity and maximum 2D diameter, meaning that HPV+ tumours were rounder and smaller, respectively, than HPV− tumours as seen in structural MRI sequences [31,57]. Also, the higher histological homogeneity of HPV+ compared to HPV− could explain the differences in the textural features, such as in the busyness and complexity [2,31].

Furthermore, the differences we found in DW-MRI are also reflected in histopathology. HPV+ tumours are more often poorly differentiated, which in turn leads to higher cell attenuation and an increased nuclear-to-cytoplasmic ratio and decreased extracellular space [58,59,60]. As ADC is a measure of the diffusion of water molecules within tissue, it can be expected that ADC is lower in the HPV+ group than in the HPV− group due to the aforementioned higher cellularity [42]. The histogram-based features of skewness and kurtosis were mentioned as useful features in several articles [2,21,22,26,31,45]. This corresponds with histological findings of HPV+ tumours being organized in homogeneous clusters with little interstitial space, leading to a higher kurtosis in ADC distribution as there is a homogeneous tumour matrix [2,61]. HPV+ tumours tend to have more outliers, since there is a small number of cells with high ADC values and a larger number of cells that are more densely packed with low ADC values [2]. This causes a higher skewness in the distribution of ADC values [2]. On the other hand, HPV− tumours show a higher heterogeneity caused by a higher prevalence of keratin pearls, intratumoural necrosis, haemorrhage, and other factors [2]. Therefore, the distribution tends to be more Gaussian, leading to a lower skewness [2,62].

To the best of our knowledge, two other systematic reviews have been published on the use of imaging to differentiate between HPV+ and HPV− cases in head and neck carcinomas. However, these studies had a different scope, as these were focused on texture analysis for HPV status determination using MRI, CT and PET or only focusing on average ADC values from DW-MRI, respectively [62,63].

The findings of our study should be considered alongside its limitations. One of the important limitations is that there was a high heterogeneity in the parameters used for DW-MRI scans. The chosen b-values varied across studies, which is expected to introduce bias in the results [29,64]. Moreover, diffusion time, which can affect ADC values, was not reported in any of the papers. In addition, the different types of fitting lead to different ADC values, whilst the different segmentation models across studies introduced more heterogeneity. Because of the high heterogeneity, we were not able to perform a pooled analysis.

Furthermore, it should be noted that we included studies that investigated OPSCC, even if other head and neck subsites were also included in the studies. Although those study populations still consisted largely of OPSCC, it is possible that their results were affected by the other subsites, and thus, this should be considered as a limitation.

Moreover, it is possible that publication bias has played a role in the included studies, as each study found at least one parameter that was different between HPV+ and HPV− tumours, or one useful predictive parameter for HPV status. This may have led to an overestimation of the effectiveness of using MRI to find differences between HPV+ and HPV− tumours or predict HPV status.

Lastly, the quality of a systematic review is inherently limited by the quality of the included studies. Even though most studies had a low bias, we found that the quality of the studies varied across the included papers. This should be taken into consideration when interpreting the results. None of the studies made use of an independent dataset for testing, thus limiting the generalizability of the findings.

## 5. Conclusions

In conclusion, our results indicate that parameters derived from structural MRI and DW-MRI show the potential to discriminate between HPV+ and HPV− tumours and predict the HPV status. However, due to the heterogeneity in the methods of studies with similar aims and an overall lack of external validation, further research is needed. At the current time, acquisition methods like DCE-MRI or ASL have yet to prove their added value to the differentiation of HPV status in OPSCC patients. So far, no predictive models have been introduced in clinical practice or used as a replacement for invasive histopathological determination methods, though using such a model alongside p16 immunohistochemistry could be considered to overcome its pitfall, e.g., false positive rates. Before introduction in clinical practice, validation on external, independent datasets is needed.

## Figures and Tables

**Table 1:** Study characteristics of included descriptive studies. ADC = apparent diffusion coefficient; DCE = dynamic contrast-enhanced; DW-MRI = diffusion-weighted magnetic resonance imaging; GOH = gradient orient histogram; GTV = gross tumour volume; H = high risk of bias; HPV = human papillomavirus; L = low risk of bias; LN = lymph node; MRI = magnetic resonance imaging; NR = not reported; OC = oral cavity; OP = oropharynx; OPSCC = oropharyngeal squamous cell carcinoma; p = prospective; PCR = polymerase chain reaction; PT = primary tumour; r = retrospective; RT = radiotherapy; S = somewhat risk of bias; v_e_ = extravascular extracellular volume fraction.

Study Characteristics						Investigated Feature Category				Significant Variables
First Author, Year	Subsite	Study Design	Total Patient Number (HPV+:HPV−)	HPV Determination Method	QUADAS-2 Score	Clinical Variables	Structural MRI	DW-MRI	Other MRI	
Cao 2019 [41]	OC, OP, larynx, hypopharynx, nasal sinonasal	p	54 (31:23)	p16	L		x	x		ADC_mean_ and GTV in PT.
Connor 2021 [50]	OP, larynx, hypopharynx	p	65 (46:19)	p16 or in situ hybridization	L			x		Increase in ADC_mean_ after 6 and 12 weeks in PT.
Connor 2022 [43]	OP, larynx, hypopharynx	p	65 (46:19)	p16 or in situ hybridization	S			x		ADC_mean_ and ADC_min_ in PT.
Chawla 2020 [35]	OP, OC, larynx, other	r	32 (21:11)	p16	L			x	x	No significant differences.
De Perrot 2017 [2]	OC and OP	r	105 (84:21)	p16 + PCR	L			x		Mean, median, 25th quartile, 75th quartile, interquartile range, skewness, excess kurtosis of ADC in PT.
Driessen 2015 [25]	OP, OC, larynx, hypopharynx.	r	73 (6:67)	p16 + PCR	L			x		ADC_mean_ in PT.
Giannitto 2020 [19]	OP	r	29 (20:9)	p16 + PCR	L		x			10th percentile and 90th percentile of GOH from T1 in PT.
Han 2018 [28]	OC and OP	r	41 (16:25)	Hybrid Capture II	L			x	x	ADC_min_ in LN.
Huang 2017 [20]	OP	r	98 (33:65)	p16	L	x	x			Alcohol consumption, cigarette smoking, betel quid chewing, necrotic nodal metastasis, cystic nodal metastasis.
Lenoir 2022 [29]	OP	r	34 (11:23)	p16 + PCR	H			x		ADC_mean_, skewness, excess kurtosis in PT.
Nakahira 2014 [38]	OP	r	26 (12:14)	p16	L			x		ADC_mean_ and ADC_min_ in PT.
Peltenburg 2020 [39]	OP, OC, larynx, hypopharynx	r	80 (18:62)	NR	L			x		ADC_mean_ in PT.
Schouten 2015 [49]	OP	r	44 (22:22)	p16 + PCR	L	x		x		Smoking, alcohol consumption, T-stage, N-stage.
Vidiri 2020 [47]	OP	r	52 (33:19)	p16 + PCR	S				x	DCE: kurtosis of v_e_ in PT.

**Table 2:** Study characteristics of included studies that aim to predict HPV status. Acc = accuracy; ADC = apparent diffusion coefficient; APT = amide proton transfer; AUC = area under the receiving operating curve; DCE = dynamic contrast-enhanced; D_t_ = tissue diffusion coefficient; DW-MRI = diffusion-weighted magnetic resonance imaging; GOH = gradient orient histogram; GLCM = grey level co-occurrence matrix; GTV = gross tumour volume; H = high risk of bias; HPV = human papillomavirus; L = low risk of bias; LN = lymph node; MRI = magnetic resonance imaging; NGTDM = neighbourhood grey tone difference matrix; NR = not reported; OC = oral cavity; OP = oropharynx; p = prospective; PCASL = pseudo-continuous arterial spin labelling; PCR = polymerase chain reaction; PT = primary tumour; r = retrospective; RT = radiotherapy; S = somewhat risk of bias; sens = sensitivity; spec = specificity.

Study Characteristics						Investigated Feature Category				Analysis Methods						
First Author, Year	Subsite	Study Design	Total Patient Number (HPV+:HPV−)	HPV Determination Method	QUADAS-2 Score	Clinical Variables	Structural MRI	DW- MRI	Other MRI	Type of Model	Type of Validation	AUC	Acc	Sens	Spec	Significant Variables
Ahn 2021 [40]	OP	p	58 (45:13)	p16 + PCR	S				x	NR	Leave one out cross-validation	0.745	0.759	0.756	0.769	PCASL: standard deviation and 95th tumour blood flow in PT.
Chan 2017 [23]	OP	p	682 (488:194)	p16	L	x	x			logistic regression	Bootstrapping on total dataset	0.84	NR	NR	NR	Border definition, exophytic, ulceration, necrosis, LN metastases, LN laterality, LN structure. Age, sex, tumour subsite, T-classification, N-classification, smoking.
Piludu 2021 [27]	OP	p	100 (69:31)	p16 + PCR	S	x	x	x	x	multivariate logistic regression	Bootstrapping on total data	0.87	0.863	0.949	0.619	ADC_mean_ and D_t_ in PT and LN. Smoking, alcohol, tumour subsite.
Vidiri 2019 [46]	OP	p	73 (54:19)	p16 + PCR	S			x		decision tree	5-fold cross-validation, no separate test set	NR	0.808	0.857	0.647	Alcohol intake, smoking. D_t_, ADC_mean_ in PT.
Bos 2021 [31]	OP	r	153 (77:76)	p16	L		x			logistic regression	60% training set: 1000 iterations of 4-fold cross-validation 40% test set: bootstrapping with 500 iterations	0.871	0.78	0.88	0.68	Sphericity, maximum 2D diameter, kurtosis, skewness, maximum wavelet in PT. Smoking, node-negative disease, tumour sublocation, T-stage.
Chan 2016 [42]	OP	r	40 (28:12)	p16	L	x		x		logistic regression	No separate validation set	0.92	NR	NR	NR	ADC_mean_ in PT and LN.
Choi 2016 [26]	OC and OP	r	22 (15:7)	p16	S				x	NR	No separate validation set	0.86	0.818	0.8	0.857	DCE in PT: K^trans^: mean, 25th, 50th, 75th percentile. k_ep_: 25th percentile.
Freihat 2021 [44]	OP	r	33 (16:17)	p16	L		x	x		multiple linear regression	No separate validation set	0.697	NR	0.769	0.733	ADC_mean_ in PT.
Fujima 2022 [37]	OP	r	31 (16:15)	NR	L		x		x	NR	No separate validation set	0.80	0.77	0.75	0.80	APT: coefficient of variation in PT.
Marzi 2022 [48]	OP	r	144 (100:44)	p16 + PCR	H	x		x		naive Bayes	Stratified 5-fold cross-validation, no test set	0.81	0.67	0.76	0.5	50th percentile of D_pt_, inverse difference moment of ADC_LN_. Tumour subsite, smoking, alcohol, sex.
Park 2022 [21]	OP	r	155 (136:19)	p16	L		x			LightGBM	80% training set: 5-fold cross-validation. 20% test set	0.83	NR	NR	NR	Contrast, total energy, energy, sphericity, dependence entropy.
Ravanelli 2018 [45]	OP	r	59 (28:31)	Hybrid Capture II	H	x	x	x		multivariate logistic regression	No separate validation set	0.944	NR	0.833	0.926	ADC_mean_ in PT, smoking, N-stage.
Sohn 2021 [22]	OP	r	62 (52:10)	p16	S		x			LASSO combined with logistic regression	69% training set: 10-fold cross-validation and bootstrapping. 31% temporal test set	0.744	0.737	0.692	0.833	First-order skewness, GLCM—informational measure of correlation 1, NGTDM-coarseness, shape-flatness from postcontrast 3D T1WI, first-order skewness, NGTDM-strength from T2WIs
Suh 2020 [32]	OP	r	60 (48:12)	p16 or PCR	L		x	x		logistic regression	Three-fold cross-validation, repeated 20 times, no separate test set.	0.77	NR	0.71	0.72	ADC in PT: entropy_std_, correlation_std_, homogeneity1_std_, entropy_std_, correlation, difference variance. T1 in PT: autocorrelation_std_.

**Table 3:** Main feature for all feature categories. ADC = apparent diffusion coefficient; D_t_ = tissue diffusion coefficient; DW-MRI = diffusion-weighted magnetic resonance imaging; MRI = magnetic resonance imaging.

Investigated Feature Category	Main Findings
Clinical variables	Tobacco use, alcohol use, tumour sublocation.
Structural MRI	Tumour size, necrosis, ulceration.
DW-MRI	ADC_mean_, D_t_.
Other MRI sequences	No widely proven features yet

**Table 4:** Characteristics of DW-MRI-specific studies. ADC = apparent diffusion coefficient; DW-MRI = diffusion-weighted magnetic resonance imaging; HPV = human papillomavirus; IQR = interquartile range; NR = not reported; std = standard deviation. x = b-value used in study.

Paper	Segmentation Method		b-Values (s/mm^2^)								ADC_mean_ (mm^2^/s)					
First Author, Year	Tumour Delineation (Slice(s) or Entire Tumour Volume)	Exclusion Necrosis or Cysts (Yes/No)	0	50	100	150	500	800	1000	Other	Average ADC_mean_ HPV+ *	std ADC_mean_ HPV+ **	Average ADC_mean_ HPV− *	std ADC_mean_ HPV− **	*p*-Value	Used Statistical Test
Descriptive Studies with DW-MRI																
Cao 2019 [41]	volume	yes		x				x			1.34 × 10^−3^	4.00 × 10^−5^	1.48 × 10^−3^	5.00 × 10^−5^	0.04	Mann–Whitney U test.
Chawla 2020 [35]	volume	yes	x				x		x		NR	NR	NR	NR	NR	Mann–Whitney U test.
Connor 2021 [36]	volume	no	x	x				x		1500	NR	NR	NR	NR	NR	Independent *t*-test or Mann–Whitney U test depending on distribution.
De Perrot 2017 [2]	slice	no	x						x		1.01 × 10^−3^	1.78 × 10^−4^	1.18 × 10^−3^	1.68 × 10^−4^	<0.001	Mann–Whitney U test.
Driessen 2016 [25]	volume	yes	x			x		x			1.33 × 10^−3^	2.67 × 10^−4^	1.74 × 10^−3^	3.38 × 10^−4^	0.005	Independent *t*-test after test for normality.
Han 2018 [28]	volume	yes	x						x		Median: 9.60 × 10^−4^	IQR: (0.87–1.03) × 10^−3^	Median: 1.06 × 10^−3^	IQR: (0.99–1.15) × 10^−3^	0.114	Independent *t*-test or Mann–Whitney U test depending on distribution.
Lenoir 2022 [29]	slice	no	x	x	x		x		x	750	9.77 × 10^−4^ ***	1.83 × 10^−4^ ***	1.12 × 10^−3^ ***	1.51 × 10^−4^ ***	0.038 ***	Mann–Whitney U test.
Nakahira 2014 [38]	slice	no	x						x		9.87 × 10^−4^	1.56 × 10^−4^	1.22 × 10^−4^	2.14 × 10^−4^	0.002	Mann–Whitney U test.
Peltenburg 2020 [39]	volume	no	x					x	x		8.1 × 10^−4^	NR	9.7 × 10^−4^	NR	<0.01	NB: mean ADC_median_. Independent *t*-test.
Schouten 2015 [49]	slice	yes	x						x	750	Median: 1.46 × 10^−3^	Range: (1.04–2.16) × 10^−3^	Median: 1.56 × 10^−3^	Range: (1.18–2.18) × 10^−3^	0.51	Mann–Whitney U test.
Vidiri 2020 [47]	NR	NR	x				x		x		NR	NR	NR	NR	NR	NR
Predictive Models with DW-MRI																
Bos 2021 [31]	volume	no								NR	NR	NR	NR	NR	NR	NR
Chan 2016 [42]	volume	yes	x						x		9.75 × 10^−4^	1.68 × 10^−4^	1.23 × 10^−3^	2.42 × 10^−4^	0.0016	Independent *t*-test.
Freihat 2021 [44]	slice	yes	x					x	x		7.58 × 10^−4^	7.00 × 10^−4^	9.05 × 10^−4^	7.40 × 10^−4^	0.001	Independent *t*-test after test for normality.
Marzi 2022 [48]	volume	no	x				x	x			NR	NR	NR	NR	NR	NR
Piludu 2021 [27]	volume	no	x	x	x	x	x	x		25; 75; 300	Median: 1.26 × 10^−3^	IQR: 0.378 × 10^−3^	Median: 1.45 × 10^−3^	IQR: 0.388	0.006	Independent *t*-test or Mann–Whitney U test depending on distribution.
Ravanelli 2018 [45]	slice	no	x						x		8.60 × 10^−4^	NR	1.05 × 10^−3^	NR	0.00168	Independent *t*-test or Mann–Whitney U test depending on distribution.
Suh 2020 [32]	volume	no	x						x		NR	NR	NR	NR	NR	NR
Vidiri 2019 [46]	volume	no	x	x	x	x	x	x		25; 75; 300	1.26 × 10^−3^	IQR: (1.06–1.47) × 10^−3^	1.58 × 10^−3^	IQR: (1.33–1.83) × 10^−3^	0.003	Mann–Whitney U test after test for normality.

* Mean unless specified otherwise. ** Standard deviation unless specified otherwise. *** Calculated from b-values 0 and 1000 mm^2^/s for generalization as these were the b-values that were most often used.

## Supplementary Materials

The following supporting information can be downloaded at: https://www.mdpi.com/article/10.3390/cancers16112105/s1. Supplementary Data S1: Search strategy; Supplementary Data S2: Results from the QUADAS-2 assessment, findings per included record.

## References

[1] Gillison M.L., Koch W.M., Capone R.B., Spafford M., Westra W.H., Wu L., Zahurak M.L., Daniel R.W., Viglione M., Symer D.E. (2000). Evidence for a causal association between human papillomavirus and a subset of head and neck cancers. J. Natl. Cancer Inst..

[2] De Perrot T., Lenoir V., Ayllón M.D., Dulguerov N., Pusztaszeri M., Becker M. (2017). Apparent Diffusion Coefficient Histograms of Human Papillomavirus-Positive and Human Papillomavirus-Negative Head and Neck Squamous Cell Carcinoma: Assessment of Tumor Heterogeneity and Comparison with Histopathology. AJNR Am. J. Neuroradiol..

[3] Ang K.K., Harris J., Wheeler R., Weber R., Rosenthal D.I., Nguyen-Tân P.F., Westra W.H., Chung C.H., Jordan R.C., Lu C. (2010). Human papillomavirus and survival of patients with oropharyngeal cancer. N. Engl. J. Med..

[4] Lewis J.S., Mirabello L., Liu P., Wang X., Dupont W.D., Plummer W.D., Pinheiro M., Yeager M., Boland J.F., Cullen M. (2021). Oropharyngeal Squamous Cell Carcinoma Morphology and Subtypes by Human Papillomavirus Type and by 16 Lineages and Sublineages. Head Neck Pathol..

[5] Marur S., Burtness B. (2014). Oropharyngeal squamous cell carcinoma treatment: Current standards and future directions. Curr. Opin. Oncol..

[6] O’Sullivan B., Brierley J., Byrd D., Bosman F., Kehoe S., Kossary C., Piñeros M., Van Eycken E., Weir H.K., Gospodarowicz M. (2017). The TNM classification of malignant tumours-towards common understanding and reasonable expectations. Lancet Oncol..

[7] Golusinski P., Corry J., Poorten V.V., Simo R., Sjögren E., Mäkitie A., Kowalski L.P., Langendijk J., Braakhuis B.J., Takes R.P. (2021). De-escalation studies in HPV-positive oropharyngeal cancer: How should we proceed?. Oral Oncol..

[8] Windon M.J., D’Souza G., Rettig E.M., Westra W.H., van Zante A., Wang S.J., Ryan W.R., Mydlarz W.K., Ha P.K., Miles B.A. (2018). Increasing prevalence of human papillomavirus-positive oropharyngeal cancers among older adults. Cancer.

[9] Bozec A., Culié D., Poissonnet G., Demard F., Dassonville O. (2021). Current Therapeutic Strategies in Patients with Oropharyngeal Squamous Cell Carcinoma: Impact of the Tumor HPV Status. Cancers.

[10] Svajdova M., Dubinsky P., Kazda T., Jeremic B. (2022). Human Papillomavirus-Related Non-Metastatic Oropharyngeal Carcinoma: Current Local Treatment Options and Future Perspectives. Cancers.

[11] Khalid M.B., Ting P., Pai A., Russo J.L., Bakst R., Chai R.L., Teng M.S., Genden E.M., Miles B.A. (2019). Initial presentation of human papillomavirus-related head and neck cancer: A retrospective review. Laryngoscope.

[12] Walline H.M., Komarck C., McHugh J.B., Byrd S.A., Spector M.E., Hauff S.J., Graham M.P., Bellile E., Moyer J.S., Prince M.E. (2013). High-risk human papillomavirus detection in oropharyngeal, nasopharyngeal, and oral cavity cancers: Comparison of multiple methods. JAMA Otolaryngol. Head Neck Surg..

[13] Kim K.Y., Lewis J.S., Chen Z. (2018). Current status of clinical testing for human papillomavirus in oropharyngeal squamous cell carcinoma. J. Pathol. Clin. Res..

[14] Duncan L.D., Winkler M., Carlson E.R., Heidel R.E., Kang E., Webb D. (2013). p16 immunohistochemistry can be used to detect human papillomavirus in oral cavity squamous cell carcinoma. J. Oral. Maxillofac. Surg..

[15] Venuti A., Paolini F. (2012). HPV detection methods in head and neck cancer. Head Neck Pathol..

[16] Wang H., Zhang Y., Bai W., Wang B., Wei J., Ji R., Xin Y., Dong L., Jiang X. (2020). Feasibility of Immunohistochemical p16 Staining in the Diagnosis of Human Papillomavirus Infection in Patients With Squamous Cell Carcinoma of the Head and Neck: A Systematic Review and Meta-Analysis. Front. Oncol..

[17] Widmann G., Henninger B., Kremser C., Jaschke W. (2017). MRI Sequences in Head & Neck Radiology—State of the Art. Rofo.

[18] Paczona V.R., Capala M.E., Deák-Karancsi B., Borzási E., Együd Z., Végváry Z., Kelemen G., Kószó R., Ruskó L., Ferenczi L. (2022). Magnetic Resonance Imaging-Based Delineation of Organs at Risk in the Head and Neck Region. Adv. Radiat. Oncol..

[19] Giannitto C., Marvaso G., Botta F., Raimondi S., Alterio D., Ciardo D., Volpe S., De Piano F., Ancona E., Tagliabue M. (2020). Association of quantitative MRI-based radiomic features with prognostic factors and recurrence rate in oropharyngeal squamous cell carcinoma. Neoplasma.

[20] Huang Y.H., Yeh C.H., Cheng N.M., Lin C.Y., Wang H.M., Ko S.F., Toh C.-H., Yen T.-C., Liao C.-T., Ng S.-H. (2017). Cystic nodal metastasis in patients with oropharyngeal squamous cell carcinoma receiving chemoradiotherapy: Relationship with human papillomavirus status and failure patterns. PLoS ONE.

[21] Park Y.M., Lim J.Y., Koh Y.W., Kim S.H., Choi E.C. (2022). Machine learning and magnetic resonance imaging radiomics for predicting human papilloma virus status and prognostic factors in oropharyngeal squamous cell carcinoma. Head Neck.

[22] Sohn B., Choi Y.S., Ahn S.S., Kim H., Han K., Lee S.K., Kim J. (2021). Machine Learning Based Radiomic HPV Phenotyping of Oropharyngeal SCC: A Feasibility Study Using MRI. Laryngoscope.

[23] Chan M.W., Yu E., Bartlett E., O’Sullivan B., Su J., Waldron J., Ringash J., Bratman S.V., Chen Y.A., Irish J. (2017). Morphologic and topographic radiologic features of human papillomavirus-related and -unrelated oropharyngeal carcinoma. Head Neck.

[24] Gaddikeri S., Gaddikeri R.S., Tailor T., Anzai Y. (2016). Dynamic Contrast-Enhanced MR Imaging in Head and Neck Cancer: Techniques and Clinical Applications. AJNR Am. J. Neuroradiol..

[25] Driessen J.P., Van Bemmel A.J.M., Van Kempen P.M.W., Janssen L.M., Terhaard C.H.J., Pameijer F.A., Willems S.M., Stegeman I., Grolman W., Philippens M.E. (2016). Correlation of human papillomavirus status with apparent diffusion coefficient of diffusion-weighted MRI in head and neck squamous cell carcinomas. Head Neck.

[26] Choi Y.S., Park M., Kwon H.J., Koh Y.W., Lee S.K., Kim J. (2016). Human Papillomavirus and Epidermal Growth Factor Receptor in Oral Cavity and Oropharyngeal Squamous Cell Carcinoma: Correlation With Dynamic Contrast-Enhanced MRI Parameters. AJR Am. J. Roentgenol..

[27] Piludu F., Marzi S., Gangemi E., Farneti A., Marucci L., Venuti A., Benevolo M., Pichi B., Pellini R., Sperati F. (2021). Multiparametric MRI Evaluation of Oropharyngeal Squamous Cell Carcinoma. A Mono-Institutional Study. J. Clin. Med..

[28] Han M., Lee S.J., Lee D., Kim S.Y., Choi J.W. (2018). Correlation of human papilloma virus status with quantitative perfusion/diffusion/metabolic imaging parameters in the oral cavity and oropharyngeal squamous cell carcinoma: Comparison of primary tumour sites and metastatic lymph nodes. Clin. Radiol..

[29] Lenoir V., Delattre B.M.A., M’Rad Y., De Vito C., de Perrot T., Becker M. (2022). Diffusion-Weighted Imaging to Assess HPV-Positive versus HPV-Negative Oropharyngeal Squamous Cell Carcinoma: The Importance of b-Values. AJNR Am. J. Neuroradiol..

[30] van Dijk L.V., Fuller C.D. (2021). Artificial Intelligence and Radiomics in Head and Neck Cancer Care: Opportunities, Mechanics, and Challenges. Am. Soc. Clin. Oncol. Educ. Book..

[31] Bos P., van den Brekel M.W.M., Gouw Z.A.R., Al-Mamgani A., Waktola S., Aerts H.J.W.L., Beets-Tan R.G.H., Castelijns J.A., Jasperse B. (2021). Clinical variables and magnetic resonance imaging-based radiomics predict human papillomavirus status of oropharyngeal cancer. Head Neck..

[32] Suh C.H., Lee K.H., Choi Y.J., Chung S.R., Baek J.H., Lee J.H., Yun J., Ham S., Kim N. (2020). Oropharyngeal squamous cell carcinoma: Radiomic machine-learning classifiers from multiparametric MR images for determination of HPV infection status. Sci. Rep..

[33] Page M.J., McKenzie J.E., Bossuyt P.M., Boutron I., Hoffmann T.C., Mulrow C.D., Shamseer L., Tetzlaff J.M., A Akl E., E Brennan S. (2021). The PRISMA 2020 statement: An updated guideline for reporting systematic reviews. BMJ.

[34] Whiting P.F., Rutjes A.W.S., Westwood M.E., Mallett S., Deeks J.J., Reitsma J.B., Leeflang M.M.G., Sterne J.A.C., Bossuyt P.M.M., QUADAS-2 Group (2011). QUADAS-2: A revised tool for the quality assessment of diagnostic accuracy studies. Ann. Intern. Med..

[35] Chawla S., Kim S.G., Loevner L.A., Wang S., Mohan S., Lin A., Poptani H. (2020). Prediction of distant metastases in patients with squamous cell carcinoma of head and neck using DWI and DCE-MRI. Head Neck..

[36] Connor S., Sit C., Anjari M., Szyszko T., Dunn J., Pai I., Cook G., Goh V. (2021). Correlations between DW-MRI and 18 F-FDG PET/CT parameters in head and neck squamous cell carcinoma following definitive chemo-radiotherapy. Cancer Rep..

[37] Fujima N., Shimizu Y., Yoneyama M., Nakagawa J., Kameda H., Harada T., Hamada S., Suzuki T., Tsushima N., Kano S. (2022). Amide proton transfer imaging for the determination of human papillomavirus status in patients with oropharyngeal squamous cell carcinoma. Medicine.

[38] Nakahira M., Saito N., Yamaguchi H., Kuba K., Sugasawa M. (2014). Use of quantitative diffusion-weighted magnetic resonance imaging to predict human papilloma virus status in patients with oropharyngeal squamous cell carcinoma. Eur. Arch. Otorhinolaryngol..

[39] Peltenburg B., Driessen J.P., Vasmel J.E., Pameijer F.A., Janssen L.M., Terhaard C.H.J., de Bree R., Philippens M.E.P. (2020). Pretreatment ADC is not a prognostic factor for local recurrences in head and neck squamous cell carcinoma when clinical T-stage is known. Eur. Radiol..

[40] Ahn Y., Choi Y.J., Sung Y.S., Pfeuffer J., Suh C.H., Chung S.R., Baek J.H., Lee J.H. (2021). Histogram analysis of arterial spin labeling perfusion data to determine the human papillomavirus status of oropharyngeal squamous cell carcinomas. Neuroradiology.

[41] Cao Y., Aryal M., Li P., Lee C., Schipper M., Hawkins P.G., Chapman C., Owen D., Dragovic A.F., Swiecicki P. (2019). Predictive Values of MRI and PET Derived Quantitative Parameters for Patterns of Failure in Both p16+ and p16- High Risk Head and Neck Cancer. Front. Oncol..

[42] Chan M.W., Higgins K., Enepekides D., Poon I., Symons S.P., Moineddin R., Weinreb I., Shearkhani O., Chen A., Beelen J. (2016). Radiologic Differences between Human Papillomavirus-Related and Human Papillomavirus-Unrelated Oropharyngeal Carcinoma on Diffusion-Weighted Imaging. ORL J. Otorhinolaryngol. Relat. Spec..

[43] Connor S., Anjari M., Burd C., Guha A., Lei M., Guerrero-Urbano T., Pai I., Bassett P., Goh V. (2022). The impact of Human Papilloma Virus status on the prediction of head and neck cancer chemoradiotherapy outcomes using the pre-treatment apparent diffusion coefficient. Br. J. Radiol..

[44] Freihat O., Tóth Z., Pintér T., Kedves A., Sipos D., Cselik Z., Lippai N., Repa I., Kovács Á. (2021). Pre-treatment PET/MRI based FDG and DWI imaging parameters for predicting HPV status and tumor response to chemoradiotherapy in primary oropharyngeal squamous cell carcinoma (OPSCC). Oral. Oncol..

[45] Ravanelli M., Grammatica A., Tononcelli E., Morello R., Leali M., Battocchio S., Agazzi G.M., Bastia M.B.d.M.e., Maroldi R., Nicolai P. (2018). Correlation between Human Papillomavirus Status and Quantitative MR Imaging Parameters including Diffusion-Weighted Imaging and Texture Features in Oropharyngeal Carcinoma. AJNR Am. J. Neuroradiol..

[46] Vidiri A., Marzi S., Gangemi E., Benevolo M., Rollo F., Farneti A., Marucci L., Spasiano F., Sperati F., Di Giuliano F. (2019). Intravoxel incoherent motion diffusion-weighted imaging for oropharyngeal squamous cell carcinoma: Correlation with human papillomavirus Status. Eur. J. Radiol..

[47] Vidiri A., Gangemi E., Ruberto E., Pasqualoni R., Sciuto R., Sanguineti G., Farneti A., Benevolo M., Rollo F., Sperati F. (2020). Correlation between histogram-based DCE-MRI parameters and 18F-FDG PET values in oropharyngeal squamous cell carcinoma: Evaluation in primary tumors and metastatic nodes. PLoS ONE.

[48] Marzi S., Piludu F., Avanzolini I., Muneroni V., Sanguineti G., Farneti A., D’urso P., Benevolo M., Rollo F., Covello R. (2022). Multifactorial Model Based on DWI-Radiomics to Determine HPV Status in Oropharyngeal Squamous Cell Carcinoma. Appl. Sci..

[49] Schouten C.S., De Graaf P., Bloemena E., Witte B.I., Braakhuis B.J.M., Brakenhoff R.H., Leemans C., Castelijns J., de Bree R. (2015). Quantitative diffusion-weighted MRI parameters and human papillomavirus status in oropharyngeal squamous cell carcinoma. AJNR Am. J. Neuroradiol..

[50] Connor S., Sit C., Anjari M., Lei M., Guerrero-Urbano T., Szyszko T., Cook G., Bassett P., Goh V. (2021). The ability of post-chemoradiotherapy DWI ADCmean and 18F-FDG SUVmax to predict treatment outcomes in head and neck cancer: Impact of human papilloma virus oropharyngeal cancer status. J. Cancer Res. Clin. Oncol..

[51] Federau C. (2017). Intravoxel incoherent motion MRI as a means to measure in vivo perfusion: A review of the evidence. NMR Biomed..

[52] Tofts P.S., Brix G., Buckley D.L., Evelhoch J.L., Henderson E., Knopp M.V., Larsson H.B., Lee T.-Y., Mayr N.A., Parker G.J. (1999). Estimating Kinetic Parameters From Dynamic Contrast-Enhanced T 1-Weighted MRI of a Diffusable Tracer: Standardized Quantities and Symbols. J. Magn. Reson. Imaging.

[53] Yankeelov T.E., Rooney W.D., Li X., Springer C.S. (2003). Variation of the relaxographic “shutter-speed” for transcytolemmal water exchange affects the CR bolus-tracking curve shape. Magn. Reson. Med..

[54] Meyer H.J., Leifels L., Hamerla G., Höhn A.K., Surov A. (2019). Associations between Histogram Analysis Parameters Derived from DCE-MRI and Histopathological Features including Expression of EGFR, p16, VEGF, Hif1-alpha, and p53 in HNSCC. Contrast Media Mol. Imaging.

[55] Sijtsema N.D., Petit S.F., Poot D.H.J., Verduijn G.M., van der Lugt A., Hoogeman M.S., Hernandez-Tamames J.A. (2021). An optimal acquisition and post-processing pipeline for hybrid IVIM-DKI in head and neck. Magn. Reson. Med..

[56] Erickson B.J., Korfiatis P., Akkus Z., Kline T.L. (2017). Machine Learning for Medical Imaging. Radiographics.

[57] Lechner M., Liu J., Masterson L., Fenton T.R. (2022). HPV-associated oropharyngeal cancer: Epidemiology, molecular biology and clinical management. Nat. Rev. Clin. Oncol..

[58] Wang J., Takashima S., Takayama F., Kawakami S., Saito A., Matsushita T., Momose M., Ishiyama T. (2001). Head and neck lesions: Characterization with diffusion-weighted echo-planar MR imaging. Radiology.

[59] Mendelsohn A.H., Lai C.K., Shintaku I.P., Elashoff D.A., Dubinett S.M., Abemayor E., St. John M.A. (2010). Histopathologic findings of HPV and p16 positive HNSCC. Laryngoscope.

[60] Cantrell S.C., Peck B.W., Li G., Wei Q., Sturgis E.M., Ginsberg L.E. (2013). Differences in imaging characteristics of HPV-positive and HPV-Negative oropharyngeal cancers: A blinded matched-pair analysis. AJNR Am. J. Neuroradiol..

[61] Just N. (2014). Improving tumour heterogeneity MRI assessment with histograms. Br. J. Cancer.

[62] Westra W.H. (2012). The morphologic profile of HPV-related head and neck squamous carcinoma: Implications for diagnosis, prognosis, and clinical management. Head Neck Pathol..

[63] Bicci E., Nardi C., Calamandrei L., Pietragalla M., Cavigli E., Mungai F., Bonasera L., Miele V. (2022). Role of Texture Analysis in Oropharyngeal Carcinoma: A Systematic Review of the Literature. Cancers.

[64] Payabvash S., Chan A., Jabehdar Maralani P., Malhotra A. (2019). Quantitative diffusion magnetic resonance imaging for prediction of human papillomavirus status in head and neck squamous-cell carcinoma: A systematic review and meta-analysis. Neuroradiol. J..

