# Supplementary material for: MRI for Differentiation between HPV-Positive and HPV-Negative Oropharyngeal Squamous Cell Carcinoma: A Systematic Review
Source: Cancers (Basel). 2024 May 31;16(11):2105. doi: 10.3390/cancers16112105 (PMC11171338; doi:10.3390/cancers16112105)
Supplement: Supplementary file 1 [file cancers-16-02105-s001.zip › cancers-3000579-supplementary.pdf]

Supplementary Data S1: search strategy

Database searched	Platform	Years of coverage	Records	Records after duplicates removed
Medline ALL	Ovid	1946 - Present	75	75
Embase	Embase.com	1971 - Present	188	121
Web of Science Core Collection*	Web of Knowledge	1975 - Present	111	45
Cochrane Central Register of Controlled Trials**	Wiley	1992 - Present	21	9
**Total**			**395**	**250**

*Science Citation Index Expanded (1975-present); Social Sciences Citation Index (1975-present); Arts & Humanities Citation Index (1975-present); Conference Proceedings Citation Index- Science (1990-present); Conference Proceedings Citation Index- Social Science & Humanities (1990-present); Emerging Sources Citation Index (2005-present)

** Manually deleted abstracts from trial registries

***Google Scholar was searched via "Publish or Perish" to download the results in EndNote.

No other database limits were used than those specified in the search strategies

<u>**Database**</u> <u>**Found records**</u>

medline **75**

(Oropharyngeal Neoplasms / OR (((oropharyn*) ADJ6 (tumor* OR tumour* OR neoplas* OR cancer* OR carcinom*))).ab,ti.) AND (exp Human Papillomavirus Viruses / OR exp Alphapapillomavirus / OR exp Papillomavirus Infections / OR (hpv OR wart-virus* OR papilloma-virus* OR papillomavirus* OR Alphapapillomavir* OR (verruca ADJ3 virus)).ab,ti.) AND (exp Magnetic Resonance Imaging / OR ((magnetic* ADJ3 resonan*) OR mri OR diffusion-weighted OR dwi OR mr-imag*).ab,ti.) AND english.la. NOT (exp animals/ NOT humans/)

embase **188**

('oropharynx tumor'/exp OR (((oropharyn*) NEAR/6 (tumor* OR tumour* OR neoplas* OR cancer* OR carcinom*))):ab,ti) AND ('Wart virus'/de OR Alphapapillomavirus/exp OR 'papillomavirus infection'/exp OR (hpv OR wart-virus* OR papilloma-virus* OR papillomavirus* OR Alphapapillomavir* OR (verruca NEAR/3 virus)):ab,ti) AND ('nuclear magnetic resonance imaging'/exp OR ((magnetic* NEAR/3 resonan*) OR mri OR diffusion-weighted OR dwi OR mr-imag*):Ab,ti) NOT [conference abstract]/lim AND [english]/lim NOT ([animals]/lim NOT [humans]/lim)

Web of science **111**

TS=(((((oropharyn*) NEAR/5 (tumor* OR tumour* OR neoplas* OR cancer* OR carcinom*)))) AND ((hpv OR wart-virus* OR papilloma-virus* OR papillomavirus* OR Alphapapillomavir* OR (verruca NEAR/2 virus))) AND (((magnetic* NEAR/2 resonan*) OR mri OR diffusion-weighted OR dwi OR mr-imag*)))

Cochrane **21**

((((oropharyn*) NEAR/6 (tumor* OR tumour* OR neoplas* OR cancer* OR carcinom*))):ab,ti) AND ((hpv OR wart-virus* OR papilloma-virus* OR papillomavirus* OR Alphapapillomavir* OR (verruca NEAR/3 virus)):ab,ti) AND (((magnetic* NEAR/3 resonan*) OR mri OR diffusion-weighted OR dwi OR mr-imag*):Ab,ti)

Supplementary Data S2

Results from the QUADAS-2 assessment, findings per included record.

Study	Risk of bias				Applicability concerns		
	patient selection	index test	reference standard	flow and timing	patient selection	index test	reference standard
Ahn 2021	low	low	high	low	low	low	low
Bos 2021	low	low	high	high	low	low	low
Cao 2019	low	low	high	low	high	low	low
Chan 2016	low	high	high	low	low	low	low
Chan 2017	low	low	high	low	low	low	low
Chawla 2020	high	high	high	low	high	low	low
Choi 2016	low	low	high	low	high	low	low
Connor 2021	low	high	high	high	high	low	low
Connor 2022	low	unclear	high	high	high	low	low
De Perrot 2017	low	low	low	low	high	low	low
Driessen 2015	low	low	low	low	high	low	low
Freihat 2021	low	high	high	low	low	low	low
Fujima 2022	unclear	high	unclear	unclear	low	low	low
Giannitto 2020	low	low	low	low	low	low	low
Han 2018	low	high	low	low	high	low	low
Huang 2017	unclear	low	high	low	low	low	low
Lenoir 2022	low	unclear	low	low	low	low	low
Marzi 2022	low	low	low	high	low	low	low
Nakahira 2014	low	high	high	low	low	low	low
Park 2022	low	low	high	high	high	low	low
Peltenburg 2020	low	unclear	unclear	unclear	high	unclear	unclear
Piludu 2021	unclear	high	low	low	low	low	low
Ravanelli 2018	low	low	low	low	low	low	low
Schouten 2015	low	low	low	low	low	low	low
Sohn 2021	low	low	high	low	low	low	low
Suh 2020	low	low	high	high	low	low	low
Vidiri 2019	high	low	low	unclear	low	low	low
Vidiri 2020	high	high	low	low	low	low	low
